# The Role of Genetic Variants in the Long Non-Coding RNA Genes *MALAT1* and *H19* in the Pathogenesis of Childhood Obesity

**DOI:** 10.3390/ncrna9020022

**Published:** 2023-03-30

**Authors:** Tatiana Pavlovna Shkurat, Manar Ammar, Olga Bocharova, Elena Teplyakova, Anzhela Aleksandrova, Ruba Ali, Leonard Lipovich

**Affiliations:** 1 Academy of Biology and Biotechnology named after D I Ivanovsky, Southern Federal University, Rostov on Don 344090, Russia; tshkurat@yandex.ru (T.P.S.); aalexsandrova@sfedu.ru (A.A.); ruba.m.ali@mail.ru (R.A.); 2 Department of Children’s Diseases No. 3, Rostov State Medical University, Rostov on Don 344022, Russia; bocharova.olga.vl@gmail.com (O.B.); elenatepl7@yandex.ru (E.T.); 3 College of Medicine, Mohammed Bin Rashid University of Medicine and Health Sciences, Dubai 505055, United Arab Emirates; leonard.lipovich@mbru.ac.ae

**Keywords:** rs3200401, rs217727, *MALAT1*, *H19*, lncRNAs, obesity, insulin resistance

## Abstract

Long non-coding RNAs (lncRNAs) play important roles in the maintenance of metabolic homeostasis. Recently, many studies have suggested that lncRNAs, such as Metastasis Associated Lung Adenocarcinoma Transcript 1 (MALAT1) and Imprinted Maternally Expressed Transcript (H19), might participate in the pathogenesis of metabolic disorders such as obesity. We conducted a case-control study with 150 Russian children and adolescents aged between 5 and 17 years old in order to assess the statistical association between the single nucleotide polymorphisms (SNPs) rs3200401 in *MALAT1* and rs217727 in *H19*, and the risk of developing obesity in this population. We further explored the possible association of rs3200401 and rs217727 with BMI Z-score and insulin resistance. The *MALAT1* rs3200401 and *H19* rs217727 SNPs were genotyped using Taqman SNP genotyping assay. The *MALAT1* rs3200401 SNP was identified as a risk factor for childhood obesity (*p <* 0.05) under the dominant and allelic models, and the CT heterozygous genotype was associated with the risk of increased BMI and with insulin resistance. The *H19* rs217727 SNP had no significant association with obesity risk (all *p >* 0.05). Our findings thus suggest that *MALAT1* SNP rs3200401 is a potential indicator of obesity susceptibility and pathogenesis in children and adolescents.

## 1. Introduction

Obesity is a chronic metabolic disorder in which an individual’s caloric intake exceeds their energy expenditure for a long period of time and consequently, the excessive energy is stored as fat. At present, obesity is a worldwide public health burden, affecting the quality of life of both adults and children [1]. Pediatric obesity has a high prevalence and persistence, which makes targeting it a key objective in most nations’ healthcare agendas [2,3]. Robust evidence demonstrates that obesity in children and adolescents acts as a risk factor for being overweight and obese in adulthood, with comorbidities that include high blood pressure, type 2 diabetes (T2D), insulin resistance, and cardiovascular diseases, in addition to the psychosocial complications reported in obese children and adolescents [4]. The etiology of obesity in children, as in adults, is caused by different environmental, genetic, and epigenetic factors [3,5].

The long non-coding RNA (lncRNA) class of human genes is the most prevalent type of non-coding RNA transcriptional unit in humans. By now, it is widely accepted that the majority of human genes do not encode proteins, and that most non-coding genes are transcribed as lncRNAs [6]. LncRNAs are commonly defined as transcripts that are longer than 200 nucleotides and have the function of RNAs without being further translated into proteins [7,8]. Recent studies have pointed towards exploring the possible regulatory roles of lncRNAs in obesity development and progression, and their effect on metabolic homeostasis, adipogenesis, and insulin function [9,10]. One of the best-characterized lncRNA, isolated early in the history of the field and since studied using a variety of methods, is the metastasis-associated lung adenocarcinoma transcript 1 (MALAT1), also referred to as NEAT2. The *MALAT1* gene is located on human chromosome 11q13, and is 8.7 kb in length. It is a highly evolutionarily conserved lncRNA, and it was initially identified in the early stage of non-small cell lung cancer, from which the name is derived [11]. The lncRNA MALAT1 functions mostly in the nucleus, and regulates gene transcription [12]. MALAT1 is involved in tumorigenesis and metastasis [11]. Recent research has reported that MALAT1 promotes lipid accumulation in the liver [13] and hyperglycemia-induced inflammation [14]. It also attenuates the function of pancreatic β cells and suppresses insulin secretion [15]. Another well-studied lncRNA gene, *H19*, is imprinted maternally expressed. *H19* is a 3.0 kb gene that is located on the human chromosome 11p15.5. H19 was the first lncRNA to be discovered in the human genome [16]. *H19* has been widely investigated due to its involvement in cell growth regulation, and has maximum expression in various tissues during embryogenesis [17]. LncRNA H19 also plays an important role in metastasis and osteoblast differentiation, through transcriptional, post-transcriptional, mRNA quality control and other epigenetic regulatory mechanisms [18,19]. Moreover, H19 participates in lipogenesis regulation, adipose tissue metabolism, and insulin sensitivity, and consequently plays a role in obesity pathogenesis [20].

The advent of genome-wide association studies (GWAS) has been essential to uncovering the contribution of non-coding genomes, including lncRNA genes, to common diseases with a genetic component, such as obesity [6,21]. Many rare and common single-nucleotide polymorphisms (SNPs) in lncRNAs have been shown to contribute to obesity susceptibility. Genetic polymorphisms in lncRNAs may influence their expression and function, and thus increase or decrease an individual’s susceptibility to the disease, to the extent that the lncRNAs are the direct causative contributors rather than mere genetic markers; this feature would help to assess disease risk and clinical prognosis [21,22]. The SNPs rs3200401 in *MALAT1* and rs217727 in *H19* are associated with increased risk of T2D [23,24] and different types of cancers [25,26]. Several studies have suggested that there is association between the SNPs rs3200401 and rs217727, and the etiological mechanisms of obesity, such as insulin dysregulation, and abnormal lipid and glucose metabolism [13,20,24]. For example, the SNP rs3200401 in *MALAT1* is associated with a high concentration of triglycerides [14]. Nonetheless, there have been no reports on the association between rs3200401 and rs217727 and the susceptibility to childhood obesity. To remedy this gap in knowledge, here we have conducted a case-control study with the approval of the bioethics committee of the Academy of Biology and Biotechnology named after D I Ivanovsky of the Southern Federal University (protocol No. 2 of 17 January 2018). We aimed to evaluate the possible associations between the SNPs rs3200401 in *MALAT1* and rs217727 in *H19*, and an individual’s risk of obesity. A further subgroup analysis was performed in order to explore the potential association between rs3200401 and rs217727 and BMI and/or insulin resistance. SNPs were genotyped in DNA samples from 150 children and adolescents from the Russian Federation. This study was motivated by the high prevalence of obesity among children in Russia [27]. Therefore, it is of great value to identify potential diagnostic markers and, in particular, potential new therapeutic targets in order to manage the epidemic of obesity.

## 2. Results

In this study, we analyzed the genotype distributions of the SNPs rs3200401 in *MALAT1* and rs217727 in *H19* in 150 children and adolescents aged between 5 and 17 years old. According to their BMI Z-score, participants were divided into two groups: the first was the insulin-sensitive control group that included 50 children and adolescents of a normal weight, and the second was the main comparison group, which included 100 obese children and adolescents. The obese group was further subdivided into two groups (50 insulin-sensitive participants, INS-S, and 50 insulin-resistant participants, INS-R) according to the HOMA-IR values.

### 2.1. Characteristics of Studied Population

The clinical features of the participants are summarized in Table 1. By conducting the Shapiro–Wilk normality test, we determined the parametric and non-parametric data based on the *p*-value of the test. When *p* > 0.05, the data were considered as parametric and have been presented as mean ± standard deviation. Otherwise, when *p* < 0.05, the data were non-parametric and expressed as a median. Accordingly, total cholesterol and LDL were compared using an ANOVA test. The TG, HDL, VLDL, AC, glucose, insulin and HOMA-IR were analyzed with the Kruskal–Wallis test. The mean ages of the participants were (10.46 ± 3.23), (10.89 ± 3.73), and (13.56 ± 2.43) in the control, INS-S, and INS-R groups, respectively. Our analysis showed no significant difference in the total cholesterol levels among the studied groups, although the levels of HDL (*p* = 0.0018), LDL (*p* = 0.0316), VLDL (*p* < 0.0001), and TG (*p* < 0.0001) significantly differed among groups. Moreover, there was a significant difference in the levels of insulin (*p* < 0.0001) and atherogenic coefficient (AC) (*p* = 0.0138), but not those of glucose (*p* = 0.3045). HOMA-IR values (*p* < 0.0001) showed significant variation among the participants, as shown in Table 1.

### 2.2. Genotype of the SNP rs3200401 in the MALAT1 Gene Has a Positive Association with the Obesity Risk

The genotype distributions of the SNP rs3200401 in the control group conformed to Hardy–Weinberg equilibrium (*p* = 0.7385). The proportions of homozygous of the major allele CC, heterozygous CT, and homozygous of the minor allele TT were 80, 18 and 2% in the control group, and 48, 46 and 6% in the obese group, respectively. Compared to the CC genotype, there was a significant difference in the frequency of CT (*p* = 0.0005) between the case and control groups. Our results confirmed the association between SNP rs3200401 in *MALAT1* and the risk of obesity (*p* = 0.0009). We evaluated the association between rs3200401 and the risk of obesity using three genetic models; dominant (CT + TT vs. CC), recessive (TT vs. CC + CT), and allelic (T vs. C). The results revealed that the SNP rs3200401 is associated with obesity in the dominant (OR (95% CI) = 4.333 (1.934–9.319), *p* = 0.0002) and allelic models (OR (95% CI) = 3.305 (1.64–6.622), *p* = 0.0004), but not in the recessive model (OR (95% CI) = 3.128 (0.4844–36.57), *p* = 0.4251); the results are shown in Table 2.

To specifically determine whether the association of SNP rs3200401 with the risk of obesity occurs because of BMI and/or insulin resistance (IR), we analyzed the three genetic models in the subgroups of (control vs. INS-S for BMI) and (INS-S vs. INS-R for IR). Results showed that the SNP rs3200401 is significantly associated with BMI Z-score (*p* = 0.0002) in the dominant and allelic models. SNP rs3200401 is also associated with insulin resistance (*p* = 0.0283). The heterozygous genotype CT showed a significant difference in BMI and IR among the studied groups. The recessive model did not show any association in either of the comparison groups. The results of subgroup analysis are clarified in Table 3.

### 2.3. Genotype of the SNP rs217727 in the H19 Gene Has a Negative Association with the Obesity Risk

No significant deviations from the Hardy–Weinberg equilibrium for SNP rs217727 in *H19* were detected in the control group (*p* = 0.9876). The proportions of homozygous of the major allele GG, heterozygous AG, and homozygous of the minor allele AA were 58.33, 35.42 and 6.25% in the control group, and 56, 38 and 6% in the obese group. Compared to the GG genotype, there was no significant difference in the frequency of the AG nor AA genotypes between the cases and controls (all *p* > 0.05). None of the three genetic models showed a significant association in the main comparison group nor in the subgroup analysis. SNP rs217727 is therefore not associated with BMI nor with insulin resistance. Results are shown in Table 4.

We also carried out an epistasis analysis of the SNPs rs3200401 in *MALAT1* and rs217727 in *H19*, but did not find a significant association, which is explicable by the lack of any known direct interaction between these two RNAs in the same complex or network.

## 3. Materials and methods

### 3.1. Ethics Statement

This study was approved by the bioethics committee of the Academy of Biology and Biotechnology named after D I Ivanovsky of the Southern Federal University (protocol No. 2 of 17 January 2018). The applied methods were carried out in compliance with the World Medical Association Declaration of Helsinki: “ethical principles for medical research involving human subjects” [28], and the articles 20, 22, 23 of the Federal Law N 323-FZ of 21 November 2011. It was also carried out “on the basics of public health protection in the Russian Federation” (as amended on 26 May 2021). According to the norms of bioethics, all parents of the children and adolescents participating in this study were previously informed in detail about the objective and content of the research, and signed a written informed consent form before donating any biological samples.

### 3.2. Study Participants and Sample Collection

This case-control study comprised 150 children and adolescents aged between 5 and 17 years old. Participants were recruited between 10 January 2018 and 31 December 2019 in the Children’s Municipal Polyclinic No. 4 in Rostov-on-Don, Russian Federation, under the guidance of the head of the department and the chief physician of the polyclinic of Rostov State Medical University. Whole blood samples were collected from participants after an overnight fasting (10–12 h).

### 3.3. Biochemical Analysis and Obesity Assessment

Biochemical analysis was carried out in the clinical and diagnostic laboratory of the medical center “Nauka” (Rostov-on-Don, Russian Federation) to measure the following: total cholesterol (TChol), triglycerides (TG), high-density lipoprotein cholesterol (HDLc), low-density lipoprotein cholesterol (LDLc), and very low-density lipoprotein cholesterol (vLDLc) concentrations. Circulating levels of glucose and insulin were also determined. Other detailed clinical information about the study population, including age and gender, are shown in Table 1.

Children and adolescents were classified into a normal weight insulin-sensitive control (n = 50) group and a total obese (n = 100) group, according to the BMI Z-score (the standard deviation from the normal body mass index of the same-age same-sex child exemplar) and as clarified by the WHO standards (underweight children < −2 SD, normal weight is between −1 and +1, overweight is between +1 and +2 and obese children are over > +2) [29,30]. This was the main group comparison of the research. Furthermore, the obese group was divided into two insulin-sensitive (INS-S) and insulin-resistant (INS-R) subgroups based on their insulin sensitivity and resistance status, which had been evaluated by calculating the homeostasis model assessment of insulin resistance (HOMA-IR). The HOMA-IR was determined by using the following formula: [fasting insulin (µLU/mL) * fasting glucose (mmol/L)/22.5].

### 3.4. SNP Selection

Using the dbSNP database (https://www.ncbi.nlm.nih.gov/snp/ (accessed on 21 September 2022)), we selected all the SNPs within the lncRNA genes *MALAT1* and *H19* with a minor allele frequency >5%. After screening the database for evidence of SNPs in *MALAT1* and *H19* genes that have an association with obesity and metabolic dysregulation, two candidate SNPs were selected: rs3200401 in *MALAT1* and rs217727 in *H19*. The rs3200401 and rs217727 had been, in other studies and populations, reported to be associated with abnormal lipid profile and metabolic disorders such as T2D [14,23,24]. 

### 3.5. DNA Extraction and Genotyping

DNA was extracted and SNPs were genotyped in the laboratory of the Genetic Institute of the Academy of Biology and Biotechnology named after D I Ivanovsky of the Southern Federal University. Genomic DNA was isolated from peripheral blood leukocytes using the thermocoagulation method and using the “DNA express blood” kit applied by Lytech Company (Moscow, Russian Federation, https://lytech.ru/ (accessed on 15 May 2018)). The single nucleotide polymorphisms rs3200401 in *MALAT1* and rs217727 in *H19* were genotyped using the TaqMan SNP Genotyping Assay in the Applied Biosystems™ QuantStudio™ 5 Real-Time PCR System (Applied Biosystems, Waltham, MA, USA) The RT-PCR reagents with specific primer/probe sets were synthesized and applied by Syntol Company (Moscow, Russia, http://www.syntol.ru/about/ (accessed on May 2022)). Primer and probe sequences are available upon request. No template sample was used as a negative control in the assays. In total, 10% of samples were chosen randomly to repeat the genotyping, and the results were 100% consistent. The reaction mixture (25 μL) contained 10 μL of 2.5 TaqMan^®^ Genotyping Master Mix (Syntol, Moscow, Russian Federation), 0.25 μL of MgCl_2_, 0.5 μL of each primer, 0.25 μL of each probe, 8.25 μL of ddH_2_O and 5 μL of genomic DNA for each SNP. For *MALAT1* rs3200401 SNP, the PCR program was set at 95 °C for 10 min; this was followed by 40 cycles at 95 °C for 15 s and 60 °C for 90 s. For *H19* rs217727, there was one cycle at 95 °C for 5 min, then 40 cycles at 95 °C for 15 s and 60 °C for 40 s.

### 3.6. Statistical Analysis

The obtained data from the biochemical analysis and genotyping were computed and analyzed by GraphPad Prism, version 8.0.1 (https://www.graphpad.com (accessed on 5 December 2018)) software. Continuous data were tested for normality by the Shapiro–Wilk test. The parametric data were presented as means ± standard deviation (SD), and then analyzed using a one-way ANOVA test. The non-parametric data were presented as a median and were compared using the Kruskal–Wallis test. The Hardy–Weinberg equilibrium (HWE) was assessed by comparing the expected frequencies with the observed genotype frequencies among the control group using a chi-squared (χ^2^) test. The differences in the distribution of the categorical variables (genotypes) in the studied groups were evaluated by Fisher’s test. The association between the genotypes and obesity risk were assessed using the odds ratio (OR) with 95% confidence interval (CI). A *p*-value < 0.05 was considered statistically significant. For the epistasis analysis of the studied polymorphisms, a Multifactor Dimensionality Reduction v.1.1.0 (MDR) software was used (www.epistasis.org/mdr.html (accessed on 24 December 2014)) [31]. MDR is used to study intergenic interactions in polygenic diseases for case-control studies. The model with the lowest prediction error and the highest reproducibility was chosen among all the obtained models. To minimize type one statistical errors in the MDR analysis, we used Bonferroni correction. The differences were considered significant if the respective *p* values were less than (pbonf = 0.006).

## 4. Discussion

As we enter the third decade of the post-genomic era, the role of the non-coding parts of the human genome in diseases is becoming progressively better elucidated. An increasing number of lncRNAs have been recently linked to multiple pathogenesis mechanisms, including those underlying cancers, and developmental and metabolic disorders, as reported in lncRNA and the Disease Database (http://www.rnanut.net/lncRNAdisease/ (accessed on 15 May 2018)) [8,22,32]. Currently, LncRNAs that participate in the regulation of the metabolic pathways that lead to obesity are being investigated. In fact, both functional and population genomics approaches have been employed to highlight numerous lncRNAs as putative contributors to obesity [9,10,33]. This includes the widely studied MALAT1 and H19 lncRNAs, previously discovered in other contexts and more recently assigned to obesity-relevant roles. Most of the studies on these two lncRNAs focus on their role in cancer [11,17]. However, there is a recent tendency towards identifying their involvement in the pathogenic mechanisms of obesity, such as fat accumulation, insulin dysfunction and impaired lipid and glucose metabolism. In mice, MALAT1 suppresses insulin secretion by inhibiting *PDX-1* expression in β cells, and MALAT1 depletion improves insulin secretion and signaling in response to glucose [34]. Another study found that MALAT1 regulates liver steatosis by promoting the accumulation of lipids in the hepatocytes [13]. *H19* is an imprinted oncofetal gene, and encodes a lncRNA transcript involved in the inhibition of adipocyte differentiation in bone marrow mesenchymal stem cells [18]. It has been recently reported that H19 regulates lipogenesis by regulating the expression level of acetyl-CoA carboxylase, fatty acid synthesis and *PPAR-Ƴ* genes [20]. Furthermore, H19 induces hepatic glucose production [35] and gluconeogenesis [36] in the liver, which promotes hyperglycemia and insulin resistance. Many human disease risk loci map to *MALAT1* and *H19*, which is consistent with the ubiquitous and high expression of these two lncRNAs in diverse tissues. In our study, we selected two candidate SNPs based on the published evidence showing that these SNPs are associated with an abnormal metabolic profile and disorders; however, the specific underlying mechanism remains to be elucidated. By searching the lncRNA SNP database (http://bioinfo.life.hust.edu.cn/lncRNASNP/ (accessed on 4 January 2018)), we found that rs3200401 in *MALAT1* and rs217727 in *H19* cause changes in the secondary structure of the lncRNA transcripts, which may lead to the loss or gain of the interactional function with other molecules. Our findings were supported by a study by Miao et al. [37], which suggested that the C > T status of rs3200401 could alter the sponging function of MALAT1 by changing its structure, which will lead to the loss of the hsa-miR–1324 miRNA binding site along this lncRNA. McCown et al. [38] demonstrated, based on RNAfold software, that rs3200401 decreases the stability of the hairpin domain of MALAT1, which means that the miR-217-5p cannot access its binding site as easily. Another study showed that *H19* rs217727 may alter a local folding structure of lncRNA H19, thus affecting the functional interactions of H19 [39]. The SNPs rs3200401 in *MALAT1* and rs217727 in *H19* are associated with an increased risk of T2D and an irregular lipid profile [23,24]. However, no research has previously been conducted to evaluate their association with an individual’s susceptibility to childhood obesity, at least among Russian children. Accordingly, we performed this case-control study with 150 children and adolescents as a pilot investigation into the association between *MALAT1* rs3200401 and *H19* rs217727, and obesity in children and adolescents aged between 5 and 17 years old. The results showed that *MALAT1* rs3200401 has a positive association with an individual’s susceptibility to obesity (*p* = 0.0009). The heterozygous CT genotype increased the risk of obesity, compared to the CC wild genotype (*p* = 0.0005). Moreover, the dominant (*p* = 0.0002) and allelic (*p* = 0.0004) genetic models revealed a significant association. To determine whether this association is more a function of increased BMI or of insulin resistance, we further divided the obese group according to their HOMA-IR values, into insulin-sensitive and insulin-resistant groups, and tested all the genetic models of rs3200401. The findings demonstrated that rs3200401 was significantly associated with BMI (*p* = 0.0002) and with insulin resistance (*p* = 0.0283). The heterozygous genotype CT of rs3200401 showed a significant difference when compared to the CC homozygous genotype of the major allele; thus, it can be considered as a risk factor for both an increased BMI and insulin resistance in children and adolescents from Russia. The CT genotype may be implicated in lipogenesis, fat accumulation, glucose metabolism, insulin secretion or signaling processes, but the molecular mechanism remains to be clarified. No effect of *H19* rs217727 on obesity susceptibility (all *p* > 0.05) was revealed. Our study admittedly has several limitations. First, the participants were enrolled from only one polyclinic and hence represented merely a single location within one city. Secondly, the sample size was very low compared to typical obesity GWAS studies, such as those of the CHARGE Consortium and its related efforts, which include thousands of samples. Thirdly, the age range of children and adolescents was not used as an independent variable or as a criterion. However, the genotype distributions of the control group respected the HWE. Fourth, obesity has a polygenic nature, with complex gene–environment interactions; therefore, environmental factors should also be considered in any integrated future risk assessment. Indeed, they might help to unravel significant patterns that are obscured in our current analysis and to functionally pinpoint links between these lncRNAs’ interactions with environmental signals and the development of obesity. Additional studies that encompass a larger sample size of mixed ethnic groups from diverse geographical regions should be performed in order to determine whether these SNPs’ minor alleles confer population-specific risks; this is a possible finding given their significance in other populations in published studies. Understanding the role of different SNPs in the pathogenesis of obesity would serve to provide new markers for diagnosis, and thus pinpoint these, and other, lncRNAs as new potential targets for use in treatment and management strategies amidst the growing interest in drug-targeting the non-coding transcriptomes.

## 5. Conclusions

In conclusion, we found that the *MALAT1* rs3200401 SNP is associated with the risk of obesity in Russian children and adolescents. The heterozygous genotype CT can be considered as a risk factor for increased BMI and insulin resistance in children. However, the rs217727 SNP in *H19* has no association with BMI nor with IR in our studied group. The specific underlying molecular mechanism remains to be investigated. Future studies are required to validate our findings in a larger sample of different populations and ethnic groups.

## Figures and Tables

**Table 1:** Characteristics of the studied groups.

	Control	INS-S	INS-R	*p*-Value
	n = 50	n = 50	n = 50	
Age	10.46 ± 3.23	10.89 ± 3.73	13.56 ± 2.43	
Gender				
Male	32	28	32	
Female	18	22	18	
Total cholesterol	3.93 ± 0.7	3.80 ± 0.8	3.84 ± 0.7	0.6551
HDL	1.41 ± 0.35	1.45 ± 0.39	1.15	0.0018
LDL	2.24 ± 0.54	1.97 ± 0.63	1.97 ± 0.61	0.0316
VLDL	0.29	0.38	0.65 ± 0.32	<0.0001
TG	0.59	0.77	1.32 ± 0.66	<0.0001
AC	1.71	1.75	2.37 ± 0.99	0.0138
Glucose	4.84	4.83 ± 0.5	4.97 ± 0.81	0.3045
Insulin	11.68 ± 3.87	16.59 ± 4.8	37.14	<0.0001
HOMA-IR	2.51 ± 0.9	3.32	7.82	<0.0001

**Table 2:** Genotype distributions and allele frequencies of SNP rs3200401 in *MALAT1* gene in control vs. total obese group.

		Obese n (%)	Controls n (%)	*p*	OR (95% CI)
Control vs. Total obese	Genotype	100	50	0.0009	
	CC	48 (48%)	40 (80%)	R ^1^	
	CT	46 (46%)	9 (18%)	0.0005	4.259 (1.93–10.18)
	TT	6 (6%)	1 (2%)	0.4251	3.128 (0.4844–36.57)
	CT + TT	52	10	0.0002	4.333 (1.934–9.319)
	Alleles				
	C	142 (71%)	89 (89%)	R	
	T	58 (29%)	11 (11%)	0.0004	3.305 (1.64–6.622)

^1^ Reference genotype/allele.

**Table 3:** Genotype distributions and allele frequencies of SNP rs3200401 in *MALAT1* gene in control vs. INS-S and INS-S vs. INS-R groups.

		INS-S n	Control n		
Control vs. INS-S	Genotype	50	50	0.0002	
	CC	20 (40%)	40 (80%)	R	
	CT	29 (58%)	9 (18%)	<0.0001	6.444 (2.61–15.21)
	TT	1 (2%)	1 (2%)	>0.9999	1 (0.05168–19.35)
	CT + TT	30	10	<0.0001	6 (2.529–14.43)
	Alleles				
	C	69 (69%)	89 (89%)	R	
	T	31 (31%)	11 (11%)	0.0008	3.635 (1.731–8.025)
		INS-R n	INS-S n		
INS-S vs. INS-R	Genotype	50	50	0.0283	
	CC	28 (56%)	20 (40%)	R	
	CT	17 (34%)	29 (58%)	0.0422	0.4187 (0.1803–0.9964)
	TT	5 (10%)	1 (2%)	0.2044	5.444 (0.6843–65.28)
	CT + TT	22	30	0.1609	0.5238 (0.2449–1.173)
	Alleles				
	C	73 (73%)	69 (69%)	R	
	T	27 (27%)	31 (31%)	0.6404	0.8232 (0.4536–1.551)

**Table 4:** Genotype distributions and allele frequencies of SNP rs217727 in *H19* gene in control vs. obese, control vs. INS-S, and INS-S vs. INS-R groups.

		Obese n	Controls n	*p*	OR (95% CI)
Control vs. Total obese	Genotype	100	48	0.9547	
	GG	56 (56%)	28 (58.33%)	R	
	GA	38 (38%)	17 (35.42%)	0.8536	1.118 (0.5305–2.317)
	AA	6 (6%)	3 (6.25%)	>0.9999	0.9574 (0.255–3.622)
	GA + AA	44	20	0.8601	1.1 (0.5542–2.264)
	Alleles				
	G	150 (75%)	73 (76.04%)	R	
	A	50 (25%)	23 (23.96%)	0.8863	1.058 (0.604–1.905)
		INS-S n	Controls n		
Control vs. INS-S	Genotype	50	48	0.7967	
	GG	26 (52%)	28 (58.33%)	R	
	GA	21 (42%)	17 (35.42%)	0.5318	1.33 (0.594–3.059)
	AA	3 (6%)	3 (6.25%)	>0.9999	0.9574 (0.2148–4.27)
	GA + AA	24	20	0.5492	1.292 (0.5659–2.755)
	Alleles				
	G	73 (73%)	73 (76.04%)	R	
	A	27 (27%)	23 (23.96%)	0.7433	1.174 (0.6147–2.289)
		INS-R n	INS-S n		
INS-S vs. INS-R	Genotype	50	50	0.7023	
	GG	30 (60%)	26 (52%)	R	
	GA	17 (34%)	21 (42%)	0.5286	0.7016 (0.3081–1.553)
	AA	3 (6%)	3 (6%)	>0.9999	1 (0.2244–4.455)
	GA + AA	20	24	0.5459	0.7222 (0.341–1.627)
	Alleles				
	G	77 (77%)	73 (73%)	R	
	A	23 (23%)	27 (27%)	0.6245	0.8076 (0.4159–1.54)

## Data Availability

The data supporting the findings of this study are available within the article.

## Abbreviations

AC	Atherogenic coefficient
BMI	Body mass index
CI	Confidence interval
GWAS	Genome-wide association studies
HDLc	High-density lipoprotein cholesterol
HOMAIR	Homeostatic Model Assessment for Insulin Resistance
IR	Insulin resistance
LDLc	Low-density lipoprotein cholesterol
LncRNA	Long non-coding RNA
